# An Urban Neo-Poverty Population-Based Quality of Life and Related Social Characteristics Investigation from Northeast China

**DOI:** 10.1371/journal.pone.0038861

**Published:** 2012-06-13

**Authors:** Fengrong Ou, Kai Li, Qian Gao, Dan Liu, Jinghai Li, Liwen Hu, Xian Wu, E. Kale Edmiston, Yang Liu

**Affiliations:** 1 The First Affiliated Hospital of China Medical University, Shenyang, Liaoning, China; 2 School of Public Health, China Medical University, Shenyang, Liaoning, China; 3 Liaoning Cancer Hospital and Institute, Shenyang, Liaoning, China; 4 Tianjin tanggu Center for Disease Control and Prevention, Tianjin, China; 5 Neuroscience Graduate Program, Vanderbilt Brain Institute, Vanderbilt University, Nashville, Tennessee, United States of America; Tehran University of Medical Sciences, Islamic Republic of Iran

## Abstract

**Objective:**

To investigate quality of life (QOL) and related characteristics among an urban neo-poverty population in northeast China, and to compare this population with a traditional poverty cohort.

**Design:**

The research was a cross-sectional survey executed from June 2005 to October 2007, with a sample of 2940 individuals ages 36 to 55 in three different industrial cities of northeast China. Data were collected on QOL status and sociodemographic characteristics. QOL was assessed using the 36-item Short Form Health Survey (Chinese version). Multiple regression analysis was employed to analyze association between sociodemographic variables and QOL.

**Results:**

The scores for QOL in the neo-poverty group were higher than those in the traditional poverty group, but lower than those in the general population. When the neo-poverty population was divided into two subgroups by age, 36–45 years and 46–55 years, the differences in QOL scores were not significant. However, there were significant differences in several dimensions between two subgroups according to unemployment time (<5 years and >5 years). Additionally, stepwise regression analysis indicated that disease burden, including disease and medical expenditures, was a common risk factor for declining QOL in the neo-poverty group.

**Conclusions:**

Despite some limitations, this study provides initial evidence that the QOL of the urban neo-poverty population lies between that of the general population and traditional poverty. QOL of the neo-poverty group approached QOL of the traditional poverty group with increased unemployment years. In addition to decreased income, disease burden is the most important factor influencing QOL status in urban neo-poverty.

## Introduction

Poverty is considered the leading risk factor or determinant for many diseases, such as AIDS and tuberculosis. Diseases resulting from poverty also perpetuate and deepen impoverishment by sapping personal and national health resources. With the wide application of biopsychosocial models in recent years, public health research has increasingly focused on the social characteristics of impoverished populations to find causes for poor health and quality of life (QOL).

Poverty is a major challenge for people in both developing and developed countries. In the World Health Organization's (WHO) report, poverty is defined not only by low income, but also by the lack of usual or socially acceptable material necessities and resources [1]. Poverty is a complex social problem; it must be understood from multiple historical and social perspectives. Studies on subpopulations of various social backgrounds and at differing historical stages are required to broaden our understanding of the dynamic relationship between poverty and health [2].

Poverty in China refers to the state of relative or absolute material deprivation, the poverty line was RMB 191 per month in Northeast China at the end of 2007 [3]. Since the1980s, China has been transitioning from a centralized to a market-based economy [4]–[6]. In northwest China, massive social change caused the emergence of a special poverty population. During asset reorganization of state-owned enterprises, many industrial workers became unemployed. These workers had little education and few skills [7]–[10]; it became difficult for them to secure reemployment. These laid-off workers form a special social group, termed the “urban neo-poverty group”, who live under the poverty line due to a lay off, are between 36 to 55 years old, lack technical skills and education, and generally have poor health and social adaptability [1], [11]–[14]. Unlike previous poverty caused by lack of security and physical limitations, this urban neo-poverty group is the result of economic transition and social structure adjustment. Their experiences reflect the impact of changing social phenomena on existing economic order and the unstable state of economic growth. Because of the social transition in China, the characteristics of this population are unique in various aspects, including health and QOL. To our knowledge, few studies have investigated poverty in this new population.

In this study, urban neo-poverty groups in three cities of northeast China were sampled. The northeast region has traditionally been an industrial center in China, similar to the previous role of the Ruhr area in Germany. During the social transition, many workers of state-owned enterprises were laid off and the number of unemployed workers in northeast China ranked among the highest in the country. The three cities chosen for study represent various types of industry in the area, including traditional heavy industry city (Shenyang), resource-exhausted city (Fuxin) and traditional light industry (Dandong).

Using the 36-item Short Form Health Survey (Chinese version) (SF-36) data of QOL survey, we investigated the effects of various factors on the QOL of the urban poverty group. The SF-36 has proven useful in comparing general and specific populations, estimating the relative burden of different diseases, differentiating the health benefits produced by a wide range of treatments, and screening individual patients. It is widely used and provides administrative and interpretation guidelines. Our investigation will contribute to the study of the social forces at work in the formation of this unique population. Furthermore, this study will help to clarify and further analyse trends of QOL in urban neo-poverty.

**Table 1:** Descriptive statistics of general conditions.

Variable	Subgroup	N	Traditional Poverty (%)	Urban neo-poverty (%)	General Population (%)
Area	Shenyang	891	100(23)	122(31)	669(32)
	Fuxin	1042	152(34)	120(30)	770(37)
	Dandong	1007	191(43)	155(39)	661(31)
Gender	Male	1873	333(75)	299(75)	1241(59)
	Female	1067	110(25)	98(25)	859(41)
Age (years)	36–45	1637	280(63)	273(69)	1084(52)
	46–55	1303	163(37)	124(31)	1016(48)
Disease	No	2233	211(48)	234(59)	1788(85)
	Yes	707	232(52)	163(41)	312(15)
Debt	No	1959	195(44)	195(49)	1569(75)
	Yes	981	248(56)	202(51)	531(25)
Household income (Yuan)	≤200	695	285(64)	223(56)	187(9)
	200–500	1373	151(34)	171(43)	1051(50)
	500–1000	626	6(2)	3(1)	617(29)
	1000–1500	136	1(0)	0(0)	135(6)
	>1500	110	0(0)	0(0)	110(5)
Educational expenditure (Yuan)	None	681	120(23)	69(17)	492(23)
	≤1000	863	223(20)	226(57)	414(20)
	1000–2000	563	52(22)	54(14)	457(22)
	>2000	832	47(35)	48(12)	737(35)
Educational Level	None	98	82(2)	5(1)	11(1)
	Primary	262	191(3)	16(4)	55(3)
	Junior	1248	68(41)	318(80)	862(41)
	Senior	715	19(31)	51(13)	645(31)
	Undergraduate	580	83(23)	7(2)	490(23)
Duration in Poverty (years)	≤5	–	–	210	–
	>5	–	–	187	–
Total	–	2940	443	397	2100

**Figure 1:**
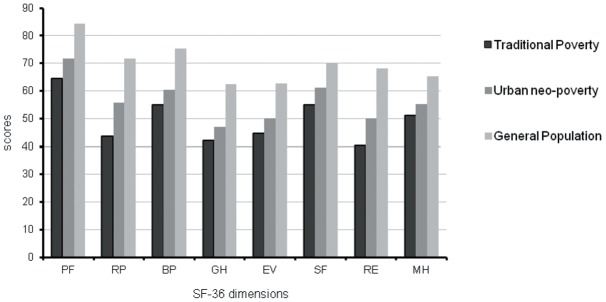
Comparison for the scores of three groups in eight SF-36 dimensions respectively, concluded that traditional poverty group and neo-poverty group were lower than general group in the eight SF-36 dimensions (p<0.05), and the scores of neo-poverty group was higher than traditional poverty group (p<0.05).

## Methods

### Sampling and data collection

Participant groups were sampled from three cities – Shenyang, Dandong, and Fuxin, which represent various types of industry in Liaoning province. Using a stratified, multiple-stage sampling method, two largely laid-off residential areas in the urban region and four general residential areas were selected for every city following the sequence of district-block-residential area. Two thousand questionnaires were distributed in each city. A face-to-face interview was scheduled once an individual was identified and agreed to participate. Each participant filled in the questionnaires by themselves, at home or in a neighborhood committee office. The interviewers included social workers and postgraduates of China Medical University, and were able to provide explanation without inducement for any unclear questionnaire items. The interview was oversaw and coordinated by a supervisor who examined the questionnaire to avoid any error and ensure quality. Each valid questionnaire was confirmed and signed by the supervisor. Both the interviewer and the supervisor were trained by an expert from Public Health School of China Medical University. The response rate was 87.5% for Shenyang (n=1750), 83.0% for Dandong (n=1660) and 92.8% for Fuxin (n=1855); the total sample comprised 5265 individuals. From this sample, we then selected people ages 36–55 as our research subjects (n=2940). All participants provided proof of residence within the city. Within this group, those who accept subsistence allowance from the government due to a lay off were defined as the neo-poverty group, and those accepting allowance due to other causes, such as diseases and debts, were defined as the traditional poverty group. During the survey period, the lowest level of subsistence allowance was 205 Yuan per month in Shenyang, 170 in Fuxin, and 196 in Dandong respectively [15].

The study was approved by the Ethics Committee at the China Medical University, and informed written consent was obtained from all participants prior to the commencement of the study.

### Measure and score

This study investigated the QOL and general status of the subjects (attached tables). The QOL of the subjects was evaluated by SF-36 [16]–[18]. Besides the Health Transition (HT) item, all items are organized into 8 scales, including Physical Functioning (PF), Role limitations due to physical health problems (RP), Bodily Pain (BP), General Health (GH), Vitality (VT), Social Functioning (SF), Role limitations due to emotional problems (RE), and Mental Health (MH) [19], [20]. The range of scores possible on each of the eight scales was from 0 to 100, with 100 representing optimal functioning as measured by the SF-36 [6], [21], [22]. Participants also completed a general conditions questionnaire, which includes basic personal and familial information, such as gender, age, health status, monthly income and expenditure, educational and medical expenses, and presence of debt, etc. All of the questionnaires were completed by the research subject regarding their current conditions.

### Statistical analysis

Internal consistency of the SF-36 items was assessed by Cronbach's α coefficient. Aggregate validity and discrimination validity were assessed by correlation analysis [23]. The QOL of different groups was analyzed and compared using the student's *t* test, multiple linear regression analysis, chi-square test, and analysis of variance. The influencing factors of each dimension of QOL were analyzed by single factor and multi-factors analysis. All analyses were conducted using SPSS 13.0 under license to the China Medical University. Mean values were given with 95% confidence intervals. A P value below 0.05 was considered significant.

**Figure 2:**
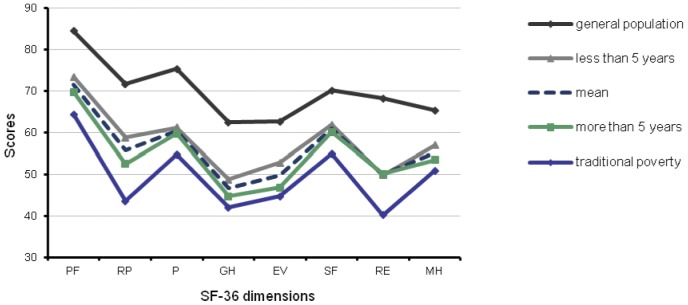
Comparison for different unemployed years of neo-poverty population with traditional poverty, mean level and general population. Less than 5 years of unemployment subgroup was above the mean level, and more than 5 years subgroup was under it. The results of BP, SF and RE dimensions in the subgroup with more than 5 years and RE dimension with less than 5 years of unemployment were very close to the scores of the traditional poverty group.

**Table 2:** Comparison of people with ≤5 and>5 years unemployment [Mean (SD)].

Duration in Unemployment (years)	N	PF	RP	BP	GH	VT	SF	RE	MH
≤5	210	73.38 (22.09)	58.81 (40.33)	61.18 (26.80)	48.79 (19.75*)	52.79 (19.76**)	61.88 (22.84)	49.84 (42.55)	56.93 (16.20*)
>	187	69.73 (25.57)	52.54 (42.95)	59.73 (28.85)	44.72 (20.39)	46.84 (20.78)	60.15 (24.36)	50.09 (42.95)	53.48 (17.03)

NOTE: *P<0.05 **P<0.01

## Results

### Descriptive statistics of general conditions

Table 1 summarizes descriptive statistics for the 2940 subjects (1873 males). The population was divided into three groups, neo-poverty (397), traditional poverty (443) and a general group (2100).

**Table 3:** Comparison of SF-36 scores between two age groups [Mean (SD)].

Age Group (years)	N	PF	RP	BP	GH	VT	SF	RE	MH
36–45	273	71.45 (24.25)	55.04 (40.90)	60.19 (27.32)	47.64 (20.92)	50.64 (20.97)	60.59 (23.54)	48.96 (41.81)	55.81 (16.92)
46–55	124	72.14 (22.97)	57.66 (43.38)	61.17 (28.81)	45.18 (18.24)	48.55 (19.22)	62.11 (23.65)	52.15 (44.63)	54.19 (16.25)

**Table 4:** Debt differences among three groups [Mean (SD)].

Group	N	Missing	Average Household Size	Per Capita Debt	Per Capita Income	Debt Per Capita Income
Urban Neo-poverty	197	200	2.7	1734.90 (3534.63)	173.65 (74.62)	12.47 (33.45)
Traditional Poverty	232	211	2.6	2312.70 (4012.57)	165.95 (100.51)	25.68 (164.63)
General Population	495	1605	2.6	3318.60 (8161.90)	687.52 (2265.87)	10.98 (34.28)

NOTE: missing refers to no debt.

### Evaluations of reliability and validity

The Cronbach's α, a reflection of reliability of the SF-36 exceeded 0.7 for all subscales (α=0.883) [22]. Set validity and discrimination validity were calculated respectively for each of the eight dimensions of SF-36. The relevant coefficient of each item after deducting the overlapping part with the dimension was higher than 0.40. The relevance between each item within the dimension was significantly higher than the relevance between each item in other dimensions, suggesting aggregate validity and discrimination validity were good.

**Table 5:** Standardized regression coefficients from multivariate stepwise regression of factors influencing QOL in three groups.

Factor	Subgroup	Physical function	Role- physical	Bodily pain	General health	Vitality	Social function	Role- emotional	Mental health
Intercept	general	89.12	65.69	69.55	58.54	57.53	69.81	64.22	55.76
	urban neo-poverty	82.97	92.45	70.15	61.48	65.00	54.74	63.75	62.72
	traditional poverty	73.93	50.41	62.68	55.38	57.51	77.12	20.18	60.55
Disease	General	−0.22***	−0.21***	−0.20***	−0.26***	−0.13***	−0.11***	−0.15***	−0.08***
	urban neo-poverty	−0.24***	−0.22***	−0.20***	−0.40***	−0.19***	−0.14**	−0.15**	−0.16**
	traditional poverty	−0.27***	−0.14**	−0.19***	−0.34***	−0.19***	−0.18***	−0.11*	−0.12**
Debt	General	−0.13***	−0.13***	−0.16***	−0.16***	−0.17***	−0.11***	−0.01***	−0.17***
	urban neo-poverty	–	−0.15**	−0.21***	–	–	−0.14**	−0.20***	–
	traditional poverty	−0.11*	−0.15**	−0.20***	−0.15**	−0.15**	−0.16***	−0.22***	−0.14**
Medical expenditure	General	−0.18***	−0.12***	−0.15***	−0.17***	−0.11***	−0.13***	−0.13***	−0.11***
	urban neo-poverty	−0.24***	−0.19***	−0.21***	−0.28***	−0.23***	−0.21***	−0.11*	−0.20***
	traditional poverty	−0.13**	−0.14**	−0.22***	−0.20***	−0.19***	−0.20***	−0.14**	−0.20***
Educational expenditure	General	0.10***	0.10***	0.05*	0.06**	–	0.11***	0.05*	0.05*
	urban neo-poverty	0.14**	–	–	0.11*	–	0.14**	–	0.10*
	traditional poverty	–	–	–	–	–	–	–	–
Household income (Yuan)	General	0.12***	0.16***	0.16***	0.14***	0.12***	0.07**	0.15***	0.12***
	urban neo-poverty	–	–	–	–	–	–	–	–
	traditional poverty	–	–	–	–	–	–	–	–
Educational level	General	0.07**	–	0.05*	0.08***	0.07**	–	–	–
	urban neo-poverty	–	–	–	–	–	–	–	–
	traditional poverty	–	–	–	–	–	–	–	–
Duration in unemployment	General	–	–	–	–	–	–	–	–
	urban neo-poverty	−0.09*	–	–	−0.11*	−0.16**	–	–	−0.11*
	traditional poverty	–	–	–	–	–	–	–	–
F Statistic	General	60.80	76.22	79.85	93.69	60.85	35.78	51.64	35.84
	urban neo-poverty	20.97	20.81	25.82	47.63	21.74	12.74	16.09	11.83
	traditional poverty	16.72	12.63	27.23	30.41	20.24	17.10	14.77	15.73
Adjusted R^2^	General	0.20	0.15	0.19	0.24	0.13	0.08	0.11	0.09
	urban neo-poverty	0.17	0.17	0.20	0.32	0.14	0.15	0.07	0.10
	traditional poverty	0.13	0.10	0.19	0.21	0.12	0.13	0.11	0.10
Degrees of freedom	General	2054	2057	2056	2055	2057	2057	2057	2056
	urban neo-poverty	392	392	392	392	393	390	394	392
	traditional poverty	437	437	437	437	438	437	437	438

NOTE: *P<0.05 **P<0.01 ***P<0.0001.

### Comparison of QOL status of residents in three groups and analysis of their characteristics

#### (1) QOL Comparison in different populations

As shown in Fig. 1, the scores on various factors of QOL in the neo-poverty group were higher than those in the traditional poverty group, but lower than in the general population.

#### (2) Association between QOL status and time of unemployment in neo-poverty group

*t* test indicated that QOL scores tended to decline with increasing unemployed years in both the neo-poverty subgroups (Table 2). The participants were divided to two subgroups according to unemployment time (<5 years and >5 years). There were significant differences in several dimensions between two subgroups, including general health, vitality, and mental health. P values were calculated using student's *t* test. According to Fig. 2, the QOL status of the neo-poverty group was between that of the general population and the traditional poverty groups. However, the QOL scores of the population with unemployment time less than 5 years approached the average level of the general population group; the results of BP, SF and RE dimensions in the subgroup with more than 5 years of unemployment were very close to the scores of the traditional poverty group.

#### (3) Comparison between different age structures in neo-poverty

We divided the neo-poverty group into two subgroups, aged 36–45 years and 46–55 years. We found the QOL status of the two subgroups in neo-poverty showed a decline with age, but this difference was not significant (Table 3). P values were calculated using student's *t* test.

#### (4) Debt differences among three groups

Debt owed by the general population was the greatest among the three groups, according to self-reported amount of debt. However, if debt amount was controlled by monthly income, the debt per Capita income in the traditional poverty group was higher than that of the other two groups. The neo-poverty group had intermediate debt when controlled for income (Table 4). P Values were calculated using analysis of variance.

### Multiple linear regression analysis for QOL status

Using stepwise multiple linear regression, we accounted for nine factors in the model: gender, age, disease, debt, length of poverty, medical expenditure, educational expenditure and educational level. The results are shown in Tables 5. All P values were calculated with multiple linear regression analysis.

In the general population group, all eight dimensions were significantly influenced by disease, debt, medical expenditure and income. The effect of educational expenditure was significant for all dimensions except for the Vitality dimension. Educational level was positively correlated with physical health measures such as status of physical function, body pain, general health and vitality.

In the neo-poverty group, disease and medical expenditure were the most influential factors across all eight dimensions. The effect of debt on physical function, pain, social function and role-emotion was significant. The influence of the dimensions of physical function, general health, vitality and mental health were attributable to unemployment years. Furthermore, educational expenditure was associated with four dimensions, including physical function, general health, social function and mental health.

In traditional poverty, all eight dimensions were significantly associated with disease, debt and medical expenditure.

## Discussion

In this study, the QOL status of the neo-poverty group was lower than control groups across all dimensions. Control groups include the general population of Hangzhou City [24], characterized by light industry and commercial development (located in the south), and a control group in the United States (data not shown) [25]. In further comparison, all eight dimensions of our measure were higher in the neo-poverty group than in the traditional poverty group.

In China, public health researchers define poverty as a broad term describing economic, social and cultural factors. These factors include low income, inability to acquire basic goods and services necessary for survival, and insufficient capacities and opportunities for a better life. Since the huge economic changes that occurred in China in the 1980s, many people, particularly the large numbers of laid-off workers from state-owned enterprises, are facing higher living costs, increased inequality, lack of resources and general uncertainty about their future. Many studies have reported that severe and persistent poverty can induce poor health, and generally adversely influence QOL [26].

The three cities involved in the study represent different industries. Shenyang is a traditional heavy industry city, mainly composed of equipment manufacturing enterprises. Fuxin was one of the first industrial energy cities since 1949. In the 1990s, as the mineral resources depleted, some mines suffered great losses; many enterprises shut down or went bankrupt, and a large number of workers were laid off. In 2000, the laid-off workers of the city totaled to 156,000, accounting for 36% of the adult population. Furthermore, according to most recent estimates, there are 180,000 people living under the poverty line, accounting for 25.3% of urban population. Dandong is a coastal light industry city with a long history with a comparatively complete category of industries.

There is a close relationship between age and QOL. Some results suggest that the status of QOL declines with age [7], [21], [27], [28], accompanied with the degeneration of multiple organs and the onset of chronic disease [29], [30]. But when the neo-poverty population was divided by age, the differences of QOL scores between the two age subgroups were not significant. Based on this effect of age on health condition, we chose to enroll only residents ages 36 to 55. Generally, after the age of 65, health conditions are largely influenced by age [31]. Our results indicate that the lower QOL scores in the neo-poverty population can be attributed to unemployment status, not age.

Our study suggests that unemployment status should be considered an important factor closely related to QOL scores in neo-poverty. In this paper, we divided the neo-poverty population into two subgroups based on duration of unemployment. The results indicate that increased length of unemployment is an important factor in declining QOL [32]. Typically, individuals in the neo-poverty population were disadvantaged before they were laid off. Because of their limited level of education, they could only perform heavy, low-skill physical labor. After lay-offs, their ability to find new employment is frequently hindered by their lack of job skills and education. QOL declines immediately with the onset of unemployment; if no appropriate intervention is made, laid-off people tend to become part of the neo-poverty population, which represents the process exhausting primitive accumulation of household wealth and social resource after unemployment.

Using stepwise regression analysis, we investigated the characteristics and interaction of various factors related with QOL score in three groups. In the general population, medical expenditure, disease, income and debt are the predominant factors that influence all eight dimensions of QOL. However, in the neo-poverty and traditional poverty populations, disease burden, including disease and medical expenditure, is a common risk factor for declining QOL. Consistent with previous studies, increased disease burden can lead to mental illness and low income [9], [31], [33], [34], which was evidenced by high disease rates in the neo-poverty (41%) and traditional poverty groups (52%) compared to the general population (15%). The individuals in the neo-poverty group and the traditional poverty group scored very low on measures of educational expenditure and level. Because score differences between these two groups were so small, these two factors, educational expenditure and culture level, were not involved in the analysis. It is well known that income level can influence QOL via standard of living [35−37], nutrition, mental stress, and educational level [38−41]. Accordingly, in this study, populations were grouped according to income level. As a result, income level was not regarded as an effective factor. Collectively, the important factors influencing the QOL in neo-poverty population are disease burden and medical expenditure.

Similar with disease burden, debt is also an important factor impacting QOL in the three studied groups. The percentage of people in debt is nearly 51% in the neo-poverty group, close to rates in the traditional poverty group (56%), and twice those of the general population (25%). Although the amount of debt in the general population is three times than of the poverty groups when accounting for mortgage and education loans, it is relatively easy for the general population to repay these debts. In the two poverty groups, although the amount of debt is comparably small, their repayment ability is poor due to their lower income. This may explain our finding of the differing effect of debt in the general population and the traditional poverty group versus the urban neo poverty group on QOL. More detailed analysis to account for this phenomenon may be needed in the future.

This study was not without limitations. Our survey data was cross sectional and only represents the living situations reported during the investigation period. However, poverty is a result of the accumulation and interaction of multiple factors across a lifetime, especially the effects of poverty on QOL. Future studies employing a longitudinal cohort will be needed to further investigate the effects of a variety of social factors on poverty and QOL across the lifetime.

This study provides initial evidence that the QOL status of an urban neo-poverty population lies between that of the general population and a traditional poverty group; QOL more closely resembles that of the traditional poverty group with increasing unemployment years. Besides decreased income, disease burden is the most important factor influencing QOL status in urban neo-poverty. Although the conditions that created the urban neo poverty group are unique to China, understanding the factors that influence QOL is increasingly important to policy design and intervention as we cope with the wave of global unemployment in our present economic depression.
